# Risk Factors of Infection, Hospitalization and Death from SARS-CoV-2: A Population-Based Cohort Study

**DOI:** 10.3390/jcm10122608

**Published:** 2021-06-13

**Authors:** Jesús Castilla, Marcela Guevara, Ana Miqueleiz, Fernando Baigorria, Carlos Ibero-Esparza, Ana Navascués, Camino Trobajo-Sanmartín, Iván Martínez-Baz, Itziar Casado, Cristina Burgui, Carmen Ezpeleta, The Working Group for the Study of COVID-19 in Navarra

**Affiliations:** 1 Instituto de Salud Pública de Navarra, 31003 Pamplona, Spain; mguevare@navarra.es (M.G.); fn.baigorria.feltrin@navarra.es (F.B.); imartinba@navarra.es (I.M.-B.); icasadob@navarra.es (I.C.); cristina.burgui.alcaide@navarra.es (C.B.); 2 Navarre Institute for Health Research (IdiSNA), 31008 Pamplona, Spain; ana.miqueleiz.zapatero@navarra.es (A.M.); ana.navascues.ortega@navarra.es (A.N.); camino.trobajo.sanmartin@navarra.es (C.T.-S.); cezpeleb@navarra.es (C.E.); 3 CIBER Epidemiología y Salud Pública (CIBERESP), 28029 Madrid, Spain; 4 Clinical Microbiology Department, Complejo Hospitalario de Navarra, 31008 Pamplona, Spain; 5 Internal Medicine Department, Complejo Hospitalario de Navarra, 31008 Pamplona, Spain; carlos.ibero.esparza@navarra.es

**Keywords:** SARS-CoV-2 infection, COVID-19, cohort study, COVID-19 hospitalization, COVID-19 severity, mortality, risk factor, epidemiology, inequality, Spain

## Abstract

We conducted a prospective population-based cohort study to assess risk factors for infection, hospitalization, and death from SARS-CoV-2. The study comprised the people covered by the Health Service of Navarre, Spain. Sociodemographic variables and chronic conditions were obtained from electronic healthcare databases. Confirmed infections, hospitalizations, and deaths from SARS-CoV-2 were obtained from the enhanced epidemiological surveillance during the second SARS-CoV-2 epidemic surge (July–December 2020), in which diagnostic tests were widely available. Among 643,757 people, 5497 confirmed infections, 323 hospitalizations, 38 intensive care unit admissions, and 72 deaths from SARS-CoV-2 per 100,000 inhabitants were observed. A higher incidence of confirmed infection was associated with people aged 15–29 years, nursing home residents, healthcare workers, people born in Latin America or Africa, as well as in those diagnosed with diabetes, cardiovascular disease, chronic obstructive pulmonary disease (COPD), chronic kidney disease, dementia, severe obesity, hypertension and functional dependence. The risk of hospitalization in the population was associated with males, higher age, nursing home residents, Latin American or African origin, and those diagnosed with immunodeficiency, diabetes, cardiovascular disease, COPD, asthma, kidney disease, cerebrovascular disease, cirrhosis, dementia, severe obesity, hypertension and functional dependence. The risk of death was associated with males, higher age, nursing home residents, Latin American origin, low income level, immunodeficiency, diabetes, cardiovascular disease, COPD, kidney disease, dementia, and functional dependence. This study supports the prioritization of the older population, nursing home residents, and people with chronic conditions and functional dependence for SARS-CoV-2 prevention and vaccination, and highlights the need for additional preventive support for immigrants.

## 1. Introduction

SARS-CoV-2 has produced more than one epidemic surge of COVID-19 during 2020 in many countries [1]. Although COVID-19 is a mild condition in most individuals, it can be life threatening for others [2]. Knowing the risk factors for infection, hospitalization and death from COVID-19 in the population may be useful for addressing clinical management, preventive measures, and vaccination programs [3]. Many studies have reported the association of sociodemographic characteristics and pre-existing conditions with severe disease and mortality from COVID-19 in clinical series or epidemiological surveillance [4,5,6,7]. Other studies have compared the characteristics of positive and negative testers [8,9]. However, studies describing risk factors for COVID-19 outcomes in the general population are scarce [10,11,12], although they are necessary to assess the risk affecting individuals in the population.

Increased odds of sociodemographic characteristics and pre-existing conditions in patients with severe COVID-19 have been reported in the first epidemic surge [13,14,15,16,17]. The low sensitivity in detecting very early cases and the limited availability of diagnostic tests in the first epidemic surge could lead to a non-representative view of the COVID-19 outcomes in the population. Between July and December 2020, there was a second epidemic surge of SARS-CoV-2 in Europe [1]. The analysis of this surge may provide a less biased view given the improvement in diagnosing cases regardless of severity and that incidence had not yet been affected by vaccination. 

The current study aimed to evaluate sociodemographic characteristics, chronic conditions and health-related variables as independent risk factors for confirmed infection, hospitalization, intensive care unit admission, and death from SARS-CoV-2 in the second epidemic surge. As the World Health Organization has proposed priority groups for vaccination that include nursing home residents, functional dependents, older age groups and individuals with certain chronic conditions [3], we also aimed to evaluate these prioritizations in the study population.

## 2. Materials and Methods

### 2.1. Study Design and Setting 

A prospective population-based cohort study was performed in Navarre, Spain, where the Health Service provides universal healthcare, free at the point of service. During the second SARS-CoV-2 epidemic surge, the wide availability of tests allowed the testing not only of all symptomatic patients and of close contacts of cases regardless of symptoms, but also the screening of population groups in specific circumstances.

The cohort included people covered by the Navarre Health Service at least from July 2019, as well as children born in Navarre after this date, so we ensured that basic medical records were available for each person. The period for prospective detection of SARS-CoV-2 infections was defined from July to December 2020. Hospitalizations and deaths from SARS-CoV-2 infections were considered in a follow-up period of 30 days after infection diagnosis. People who had been confirmed for SARS-CoV-2 infection before July 2020 were removed from the cohort.

### 2.2. Variables

The outcomes of interest were SARS-CoV-2 confirmed infection, hospitalization, intensive care unit admission and death.

Confirmed cases were defined as patients who tested positive for SARS-CoV-2 by commercial tests based on reverse transcription quantitative real-time polymerase chain reaction or antigen test in a respiratory tract sample. The antigen test was used in symptomatic patients within 5 days of the COVID-19 symptom onset [18].

COVID-19 hospitalized cases included those admitted for 24 h or more and those who died in the emergency room before admission. Deaths were obtained from electronic medical records and the mortality registry. As part of the epidemiological surveillance, medical doctors reviewed hospital admissions and deaths to identify those related to COVID-19, and only those were considered for the present study.

Sociodemographic characteristics, chronic conditions and other health-related variables at baseline were obtained from the electronic medical records. This source of information has demonstrated high sensitivity and specificity to detect chronic medical conditions [19].

Sociodemographic variables included sex, age group (0–14, 15–29, 30–49, 50–59, 60–69, 70–79 and ≥80 years old), nursing home residence, healthcare work, place of birth (Spain, Europe, Latin America, North Africa, sub-Saharan Africa, and others), place of residence (<5000, 5000–50,000, and >50,000 inhabitants), and annual taxable income level in four categories.

Major chronic conditions considered were: immunodeficiency (primary immunodeficiency, HIV infection or transplant recipient), diabetes, cardiovascular disease, chronic obstructive pulmonary disease (COPD), asthma, chronic kidney disease, cerebrovascular disease, liver cirrhosis, dementia, hematological malignancy, non-hematological cancer, severe obesity (body mass index ≥ 40 kg/m^2^), and hypertension. The lack of registered diagnosis of chronic disease was considered as not having that condition. 

From the electronic medical records, we also obtained the history of hospitalization in the prior 12 months, the smoking status (non-smoker, former smoker, current smoker, and unknown), and the functional dependence (Barthel’s index <40) [20].

### 2.3. Statistical Analysis 

The database was anonymized before the analysis. The cumulative incidence of SARS-CoV-2 confirmed infection, hospitalization, intensive care unit admission, and death per 100,000 inhabitants was calculated for each category of the analyzed variables. Poisson regression models were used to assess the independent effect of each variable for the analyzed outcomes. For every variable, the sex- and age-adjusted relative risk (RR) and the fully adjusted RR with their 95% confidence intervals (CI) were calculated. *p*-values < 0.05 were considered statistically significant. 

The population was categorized in hierarchical categories for COVID-19 vaccination priority in the following order: nursing home residents, functional dependents, and age groups starting from the oldest and split into two categories according to the presence or not of any major chronic condition. The proportion and the risk of each COVID-19 outcome were calculated in each category.

### 2.4. Ethical Aspects 

This study was approved by the Ethical Committee for Clinical Research of Navarre, which waived the requirement of obtaining informed consent (approval code: PI2020/45).

## 3. Results

### 3.1. Cumulative Incidence by Population Characteristics

**Figure 1:**
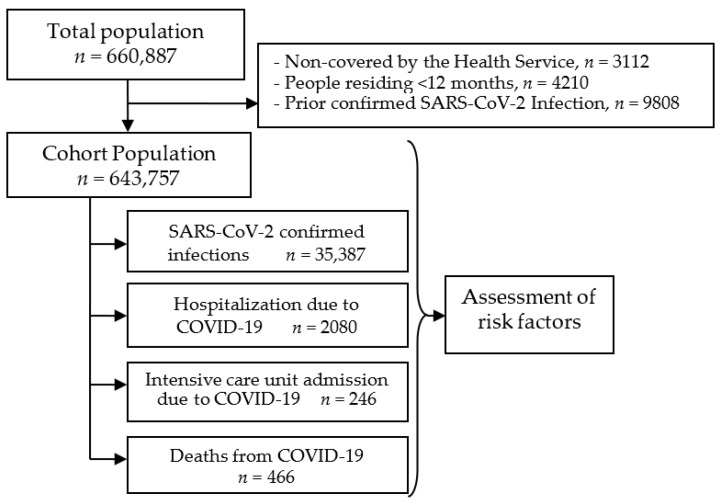
Scheme of the study.

The cohort included 643,757 people: 35,387 of them were confirmed for SARS-CoV-2 infection in the study period, 2080 were hospitalized, 246 were admitted to the intensive care unit, and 466 died from COVID-19 (Figure 1). These figures supposed cumulative incidences of 5497, 323, 38, and 72 per 100,000 inhabitants, respectively. The infections confirmed in the study period were 72% of all SARS-CoV-2 infections confirmed during the first 12 months of the pandemic.

The cumulative incidence of SARS-CoV-2 infection was high in all population groups, ranging from 3.6% in people aged 70–79 years to 13.8% in nursing home residents, followed by people born in Latin America (11.2%) or North Africa (7.6%), people with dementia (7.4%) and functional dependence (7.4%), and people aged 15–29 years (7.6%) (Table 1).

The cumulative incidence of hospitalization, intensive care unit admission and death by COVID-19 showed important differences among population groups. The highest risk of hospitalization was observed in nursing home residents (3.3%), followed by people with functional dependence (2.5%), dementia (2.2%), or aged 80 years and older (1.5%). The highest risk of intensive care unit admission was observed in people with severe obesity (191 per 100,000), liver cirrhosis (133 per 100,000), and aged 70–79 years (127 per 100,000). The highest risk of mortality from COVID-19 was found in nursing home residents (2.3%), functional dependents (2.1%), and persons with dementia (1.7%) or aged 80 years and over (0.9%).

### 3.2. Predictive Factors for Infection, Hospitalization and Severe Outcomes

The fully adjusted RR of SARS-CoV-2 confirmed infection in the population was significantly higher in people aged 15–29 years, nursing home residents, healthcare workers, people born in Latin America, North Africa or sub-Saharan Africa, people residing in municipalities of 5000–50,000 inhabitants, as well as in those diagnosed with diabetes, cardiovascular disease, COPD, chronic kidney disease, dementia, severe obesity, hypertension and functional dependence (Table 1). 

Hospitalization with COVID-19 in the population was independently associated with males, higher age, nursing home residents, people born in Latin America, North Africa or sub-Saharan Africa, those with very low income level, residence in municipalities >5000 inhabitants and hospitalization in the prior 12 months, as well as with people diagnosed with immunodeficiency, diabetes, cardiovascular disease, COPD, asthma, chronic kidney disease, cerebrovascular disease, liver cirrhosis, dementia, severe obesity, hypertension and functional dependence (Table 2).

The fully adjusted RR of intensive care unit admission for COVID-19 in the population was statistically significantly higher in males, older age up to 70–79 years, people born in Latin America or North Africa, people residing in municipalities of 5000–50,000 inhabitants, and those diagnosed with asthma, severe obesity and hypertension (Table 3).

An increased risk of death from COVID-19 in the population was independently observed in males, higher ages, nursing home residents, people born in Latin America, those with very low and low incomes, and those hospitalized in the prior 12 months, as well as in people with immunodeficiency, diabetes, cardiovascular disease, COPD, chronic kidney disease, dementia and functional dependence (Table 4).

Current smokers, but not former smokers, had a significantly lower risk of SARS-CoV-2 confirmed infection, hospitalization, and intensive care unit admission for COVID-19.

### 3.3. Assessing Priority Groups for Vaccination 

Regardless of other variables, nursing home residents and functional dependents presented the highest risks of COVID-19 hospitalization and death. Outside of these groups, aging was associated with an increased risk of hospitalization and death. In every age group, people with major chronic conditions had a higher risk of hospitalization and death. For some age groups, the presence of major chronic conditions increased the risk more than being 10 years older (Table 5). The vaccination of nursing home residents, people with functional dependence and people aged 80 years and over will cover the population groups in which 79% of deaths by COVID-19 occurred, but only those that give rise to 31% of hospitalizations and 8% of intensive care unit admissions. Extending vaccination to all people aged 50 years and over will cover the population in which 79% of hospitalizations, 87% of intensive care unit admissions and 99% of deaths from COVID-19 occurred (Table 5).

## 4. Discussion

The present population-based cohort study shows important differences in the incidence of COVID-19 hospitalizations and severe outcomes according to the characteristics of the individuals that lead to defining high-risk groups. Many of these findings are consistent with the increased risk of severe outcomes among COVID-19 cases that have been associated with specific conditions [13,14,15,16,17]. We also provide population-based information on possible differences in the risk of infection due to susceptibility or increased exposure to SARS-CoV-2 infection. Therefore, we show a complete perspective to assess the priority groups for healthcare and preventive interventions in the population. 

Since the first pandemic surge, protocols were implemented to prevent cases in nursing homes [21]; however, people residing in these facilities still presented a three-fold higher risk of infection than other people with similar characteristics did in the second surge, demonstrating the exceptional difficulties for preventing transmission in these places. The excess risk in nursing home residents was similar for SARS-CoV-2 infection and severe outcomes, suggesting that the excess risk for greater severity was due to the increased risk of infection, but not due to late or worse medical care.

Age was a very important risk factor for the outcomes evaluated. The highest risk for SARS-CoV-2 infection was observed in the group aged 15–29 years that had been less affected in the first surge due to the early closure of educational centers [7]. The risk of hospitalization for COVID-19 increased progressively with age, admission to intensive care units increased up to the age group of 70–79 years, and the risk of death rose exponentially with age. Although males did not show a higher incidence of confirmed infection [22], consistent with the literature, they presented a higher risk of hospitalization and severe outcomes, indicating their worse prognosis for this infection [17,23]. Healthcare workers presented an excess of confirmed infection but did not present excess hospitalization or severe outcomes, suggesting timely and effective medical care.

Compared to natives, people born in Latin America and Africa showed a higher risk of confirmed infection, hospitalization and severe outcomes. Possible explanations of these findings are their frequent work as caregivers or in other socially exposed activities, greater number of cohabitants, greater use of public transport, and possibly, worse access to health promotion, preventive measures and early diagnosis. A higher susceptibility related to ethnicity has also been suggested [24], but this variable was not available in the present study. Regardless of the explanation, specific interventions are urgently needed to reduce this excess risk.

Residents in municipalities of more than 5000 inhabitants presented an increased risk of SARS-CoV-2 infection that was probably related to increased social interaction. This excess risk was also observed for hospitalization admission by COVID-19. Very low- and low-income levels were risk factors for SARS-CoV-2 confirmed infection, hospitalization and mortality in the analysis only adjusted for sex and age. The association with COVID-19 mortality remained in the fully adjusted analysis, suggesting a possible delay in access to medical care.

Current smokers showed a lower risk of diagnosed SARS-CoV-2 infection and hospitalization, but they did not have a lower risk of COVID-19 mortality. These results should be considered carefully due to the high proportion of missing values in smoking status. Nevertheless, similar findings have been found in other studies [8,10,25]. These results offer a different perspective from studies reporting that smoking is associated with increased severity in COVID-19 patients [14,26]. More studies are needed to clarify the effect of tobacco on SARS-CoV-2 transmission [24,27].

The higher risk of SARS-CoV-2 infection associated with some chronic conditions, such as diabetes, cardiovascular disease, COPD, chronic kidney disease, dementia, severe obesity, hypertension and functional dependence, is especially concerning because chronic conditions also increase the risk of severe illness in the case of SARS-CoV-2 infection [7]. These conditions may increase the susceptibility to infection, and chronic patients could be exposed to infection from caregivers or in visits to healthcare centers.

Our results are consistent with many other studies showing the increased risk of severe COVID-19 outcomes among patients with major chronic conditions [13,14,15,16,17,28]. Almost all major chronic conditions were independent risk factors for COVID-19 hospitalization; asthma, severe obesity and hypertension were also related to intensive care unit admission; and several major chronic conditions were risk factors for COVID-19 mortality. However, the increased risk associated with major chronic comorbidities was not greater than the risk associated with increasing one or two decades of age.

Hypertension was independently associated with SARS-CoV-2 infection, hospitalization and intensive care unit admission, as has been reported in other studies [29], but this is in contrast with results from the same region in the first epidemic surge when hypertension was not an independent risk factor in the analysis adjusted for hypertension-related comorbidities [30].

The main strengths of our study are that we evaluated four COVID-19 outcomes using a prospective population-based cohort design and that only laboratory-confirmed cases were considered in a period with high availability of tests. Information was obtained from administrative and clinical records before the beginning of the follow-up to prevent information bias.

Some limitations should also be mentioned. Comorbidity severity and treatments, clinical manifestations of COVID-19, and the treatment received at the hospital were not available. A positive antigen test was considered confirmatory in patients with symptoms since the specificity of this test has been proved high in these cases [31]. Predictors for severe COVID-19 outcomes may be different in other places and other epidemic surges, especially after the introduction of the SARS-CoV-2 vaccine. Temporary residents and non-resident immigrants were not included in this study. Although they are a small proportion of the population, this exclusion may have affected the results.

## 5. Conclusions

These results support the prioritization of preventive interventions and COVID-19 vaccination programs in nursing home residents, people with functional dependence, older populations, and those with chronic conditions because they have a higher risk of severe outcomes than the rest of the population. Healthcare workers were at a higher risk of infection, but not for severe outcomes. Since people born in Latin America and Africa were at higher risk of infection and severe outcomes, they may need specific preventive interventions, better access to healthcare, and priority in vaccination programs.

## Figures and Tables

**Table 1:** Association between potential predictive factors and confirmed SARS-CoV-2 infection in the general population cohort.

	Infections		Sex- and Age-Adjusted Analysis			Fully Adjusted Analysis *		
	*n*	Cases per 100,000	RR	95% CI	*p* Value	RR	95% CI	*p* Value
**Total**	35,387	5497						
**Sex**								
Female	18,215	5609	1			1		
Male	17,172	5383	0.95	0.93–0.97	<0.001	0.98	0.96–1.00	0.078
**Age, years**								
0–14	5625	5457	0.99	0.95–1.02	0.441	1.01	0.97–1.05	0.526
15–29	7640	7611	1.37	1.33–1.42	<0.001	1.28	1.24–1.33	<0.001
30–49	10,248	5544	1.00	0.97–1.03	0.976	0.96	0.93–0.99	0.017
50–59	5187	5541	1			1		
60–69	2986	4204	0.76	0.72–0.79	<0.001	0.75	0.72–0.79	<0.001
70–79	1899	3557	0.64	0.61–0.68	<0.001	0.59	0.56–0.62	<0.001
80+	1802	4818	0.86	0.82–0.91	<0.001	0.64	0.59–0.68	<0.001
**Nursing home resident**	681	13,830	3.28	3.02–3.55	<0.001	3.24	2.98–3.53	<0.001
**Healthcare worker**	692	6290	1.11	1.03–1.20	0.005	1.23	1.14–1.33	<0.001
**Place of birth**								
Spain	26,779	4959	1			1		
Europe	1049	4114	0.80	0.75–0.85	<0.001	0.81	0.76–0.86	<0.001
Latin America	5738	11,175	2.11	2.04–2.17	<0.001	2.08	2.01–2.14	<0.001
North Africa	1213	7586	1.45	1.36–1.53	<0.001	1.44	1.36–1.53	<0.001
Sub-Saharan Africa	459	6387	1.23	1.13–1.35	<0.001	1.21	1.10–1.32	<0.001
Other	149	3951	0.75	0.64–0.88	0.001	0.75	0.64–0.88	0.001
**Place of residence**								
>50,000 inhabitants	11,249	5548	1.06	1.03–1.09	<0.001	1.01	0.99–1.04	0.355
5000–50,000 inhabitants	12,711	5708	1.07	1.05–1.10	<0.001	1.04	1.02–1.07	0.001
<5000 inhabitants	11,427	5234	1			1		
**Income level**								
Very low	1734	6201	1.16	1.10–1.22	<0.001	1.00	0.95–1.05	0.929
Low	20,437	5760	1.10	1.08–1.13	<0.001	0.99	0.97–1.02	0.521
Middle	12,983	5064	1			1		
High	233	5080	0.98	0.86–1.11	0.747	0.99	0.87–1.13	0.922
**Smoking status**								
Never smoker	3191	3884	1			1		
Current smoker	6119	5788	0.63	0.60–0.66	<0.001	0.67	0.64–0.70	<0.001
Former smoker	1235	5136	0.98	0.92–1.04	0.445	1.01	0.95–1.07	0.785
Unknown	24,842	5752	0.88	0.85–0.90	<0.001	0.87	0.85–0.90	<0.001
**Hospitalization in prior year**	1917	5718	1.13	1.08–1.18	<0.001	1.09	1.04–1.14	0.001
**Immunodeficiency**	267	5501	1.04	0.93–1.18	0.487	1.00	0.89–1.13	0.984
**Diabetes**	1893	4992	1.14	1.08–1.19	<0.001	1.06	1.01–1.11	0.024
**Cardiovascular disease**	2736	5216	1.07	1.03–1.12	0.001	1.08	1.03–1.12	<0.001
**COPD**	1404	5074	1.04	0.99–1.10	0.112	1.10	1.04–1.16	0.001
**Asthma**	2330	5535	0.97	0.93–1.01	0.162	1.00	0.96–1.04	0.969
**Chronic kidney disease**	989	5130	1.16	1.08–1.24	<0.001	1.11	1.04–1.19	0.002
**Cerebrovascular disease**	470	4868	1.10	1.00–1.21	0.048	0.99	0.90–1.09	0.841
**Liver cirrhosis**	632	5244	1.11	1.03–1.21	0.008	1.06	0.98–1.15	0.127
**Dementia**	369	7420	1.72	1.54–1.92	<0.001	1.25	1.11–1.40	<0.001
**Hematological malignancy**	110	4073	0.85	0.70–1.02	0.087	0.87	0.72–1.05	0.139
**Non-hematological cancer**	1695	4363	0.96	0.91–1.01	0.090	0.98	0.93–1.03	0.454
**Severe obesity**	527	6295	1.24	1.13–1.35	<0.001	1.18	1.08–1.29	<0.001
**Hypertension**	4543	4666	1.07	1.03–1.12	<0.001	1.05	1.01–1.09	0.013
**Functional dependence**	339	7399	1.65	1.48–1.85	<0.001	1.22	1.08–1.38	0.001

COPD, chronic obstructive pulmonary diseases; RR, relative risk; CI, confidence interval, * Adjusted for all the variables in the table.

**Table 2:** Association between potential predictive factors and COVID-19 hospitalization in the general population cohort.

	Hospitalizations		Sex- and Age-Adjusted Analysis			Fully Adjusted Analysis *		
	*n*	Cases per 100,000	RR	95% CI	*p* Value	RR	95% CI	*p* Value
**Total**	2080	323						
**Sex**								
Female	1000	308	1			1		
Male	1080	339	1.27	1.16–1.38	<0.001	1.32	1.21–1.45	<0.001
**Age, years**								
0–14	24	23	0.06	0.04–0.09	<0.001	0.07	0.04–0.10	<0.001
15–29	48	48	0.12	0.09–0.17	<0.001	0.11	0.08–0.15	<0.001
30–49	368	199	0.51	0.44–0.59	<0.001	0.48	0.41–0.55	<0.001
50–59	365	390	1			1		
60–69	353	497	1.28	1.10–1.48	0.001	1.25	1.07–1.45	0.004
70–79	365	684	1.77	1.53–2.05	<0.001	1.49	1.27–1.75	<0.001
80+	557	1489	3.95	3.46–4.51	<0.001	2.42	2.04–2.87	<0.001
**Nursing home resident**	162	3290	3.56	3.00–4.22	<0.001	3.23	2.69–3.88	<0.001
**Healthcare worker**	22	200	0.76	0.50–1.16	0.199	0.98	0.64–1.51	0.936
**Place of birth**								
Spain	1640	304	1			1		
Europe	62	243	1.30	1.01–1.69	0.043	1.27	0.98–1.64	0.075
Latin America	296	576	3.70	3.24–4.23	<0.001	3.47	3.02–3.99	<0.001
North Africa	53	331	2.22	1.68–2.94	<0.001	2.17	1.63–2.89	<0.001
Sub-Saharan Africa	21	292	1.86	1.20–2.87	0.005	1.63	1.05–2.54	0.029
Other	8	212	1.30	0.65–2.60	0.463	1.28	0.64–2.57	0.489
**Place of residence**								
>50,000 inhabitants	723	357	1.17	1.05–1.30	0.004	1.14	1.02–1.27	0.019
5000–50,000 inhabitants	695	312	1.20	1.08–1.34	0.001	1.16	1.04–1.29	0.007
<5000 inhabitants	662	303	1			1		
**Income level**								
Very low	102	365	2.04	1.66–2.52	<0.001	1.27	1.02–1.58	0.034
Low	1288	363	1.28	1.16–1.41	<0.001	1.05	0.95–1.16	0.372
Middle	677	264	1			1		
High	13	283	1.08	0.62–1.86	0.796	1.11	0.64–1.92	0.715
**Smoking status**								
Never smoker	191	233	1			1		
Current smoker	611	578	0.54	0.45–0.64	<0.001	0.54	0.46–0.65	<0.001
Former smoker	181	753	1.05	0.89–1.24	0.580	1.02	0.86–1.21	0.798
Unknown	1097	254	0.86	0.77–0.96	0.006	0.84	0.76–0.94	0.002
**Hospitalization in prior year**	243	725	1.52	1.33–1.74	<0.001	1.28	1.11–1.47	0.001
**Immunodeficiency**	36	742	2.04	1.47–2.84	<0.001	1.67	1.20–2.32	0.003
**Diabetes**	408	1076	1.61	1.43–1.80	<0.001	1.33	1.18–1.49	<0.001
**Cardiovascular disease**	411	784	1.33	1.19–1.50	<0.001	1.18	1.05–1.33	0.007
**COPD**	195	705	1.29	1.11–1.50	0.001	1.30	1.11–1.51	0.001
**Asthma**	147	349	1.29	1.09–1.53	0.003	1.27	1.07–1.50	0.006
**Chronic kidney disease**	275	1426	1.65	1.43–1.89	<0.001	1.41	1.23–1.63	<0.001
**Cerebrovascular disease**	135	1398	1.58	1.32–1.89	<0.001	1.27	1.06–1.52	0.011
**Liver cirrhosis**	105	871	1.66	1.36–2.02	<0.001	1.42	1.17–1.74	0.001
**Dementia**	108	2172	1.89	1.54–2.32	<0.001	1.28	1.02–1.59	0.032
**Hematological malignancy**	24	889	1.40	0.94–2.10	0.099	1.38	0.92–2.06	0.119
**Non-hematological cancer**	255	656	0.97	0.85–1.11	0.651	0.96	0.84–1.11	0.605
**Severe obesity**	79	944	2.20	1.75–2.75	<0.001	1.79	1.42–2.25	<0.001
**Hypertension**	840	863	1.27	1.15–1.41	<0.001	1.11	1.01–1.25	0.040
**Functional dependence**	116	2532	2.28	1.87–2.79	<0.001	1.54	1.24–1.91	<0.001

COPD, chronic obstructive pulmonary diseases; RR, relative risk; CI, confidence interval; *Adjusted for all the variables in the table.

**Table 3:** Association between potential predictive factors and intensive care unit admission for COVID-19 in the general population cohort.

	Intensive Care Unit Admissions		Sex- and Age-Adjusted Analysis			Fully Adjusted Analysis *		
	*n*	Cases per 100,000	RR	95% CI	*p* Value	RR	95% CI	*p* Value
**Total**	246	38						
**Sex**								
Female	92	28	1			1		
Male	154	48	1.79	1.38–2.31	<0.001	2.02	1.53–2.66	<0.001
**Age, years**								
0–14	1	1	0.02	0.00–0.11	<0.001	0.02	0–0.14	<0.001
15–29	2	2	0.03	0.01–0.13	<0.001	0.03	0.01–0.11	<0.001
30–49	30	16	0.26	0.17–0.40	<0.001	0.23	0.15–0.37	<0.001
50–59	59	63	1			1		
60–69	72	101	1.62	1.15–2.29	0.006	1.73	1.21–2.46	0.003
70–79	68	127	2.07	1.46–2.93	<0.001	2.21	1.49–3.29	<0.001
80+	14	37	0.64	0.36–1.15	0.139	0.72	0.37–1.38	0.320
**Nursing home resident**	4	81	1.47	0.54–4.01	0.455	2.07	0.75–5.74	0.161
**Healthcare worker**	4	36	1.10	0.41–2.99	0.850	1.55	0.56–4.23	0.397
**Place of birth**								
Spain	175	32	1			1		
Europe	7	27	1.31	0.61–2.80	0.491	1.24	0.57–2.67	0.588
Latin America	55	107	6.73	4.88–9.30	<0.001	6.15	4.34–8.72	<0.001
North Africa	7	44	2.84	1.32–6.10	0.008	2.88	1.30–6.36	0.009
Sub-Saharan Africa	2	28	1.67	0.41–6.80	0.472	1.38	0.34–5.70	0.654
Other	0	0	NE			NE		
**Place of residence**								
>50,000 inhabitants	84	41	1.55	1.11–2.17	0.010	1.39	0.99–1.95	0.061
5000–50,000 inhabitants	104	47	1.97	1.43–2.71	<0.001	1.80	1.30–2.50	<0.001
<5000 inhabitants	58	27	1			1		
**Income level**								
Very low	17	61	2.79	1.66–4.70	<0.001	1.49	0.85–2.61	0.162
Low	131	37	1.21	0.93–1.59	0.158	0.96	0.72–1.27	0.755
Middle	96	37	1			1		
High	2	44	1.08	0.27–4.38	0.917	1.15	0.28–4.67	0.846
**Smoking status**								
Never smoker	30	37	1			1		
Current smoker	67	63	0.50	0.32–0.77	0.002	0.57	0.36–0.89	0.014
Former smoker	27	112	0.97	0.62–1.53	0.895	1.01	0.64–1.60	0.961
Unknown	122	28	0.72	0.52–0.98	0.037	0.77	0.56–1.05	0.099
**Hospitalization in prior year**	16	48	0.89	0.54–1.49	0.662	0.84	0.50–1.41	0.516
**Immunodeficiency**	5	103	1.93	0.80–4.69	0.145	1.66	0.68–4.06	0.267
**Diabetes**	46	121	1.56	1.11–2.17	0.009	1.21	0.86–1.72	0.276
**Cardiovascular disease**	33	63	1.00	0.69–1.47	0.988	0.90	0.61–1.33	0.595
**COPD**	22	80	1.14	0.73–1.78	0.559	1.22	0.78–1.92	0.386
**Asthma**	23	55	1.94	1.26–2.99	0.003	1.84	1.19–2.83	0.006
**Chronic kidney disease**	22	114	1.70	1.07–2.68	0.025	1.49	0.94–2.39	0.093
**Cerebrovascular disease**	7	73	0.89	0.42–1.91	0.774	0.85	0.40–1.83	0.679
**Liver cirrhosis**	16	133	1.72	1.03–2.86	0.037	1.43	0.85–2.39	0.173
**Dementia**	0	0	NE			NE		
**Hematological malignancy**	1	37	0.52	0.07–3.72	0.516	0.55	0.08–3.91	0.548
**Non-hematological cancer**	29	75	0.87	0.59–1.30	0.506	0.92	0.61–1.37	0.673
**Severe obesity**	16	191	3.69	2.22–6.13	<0.001	3.05	1.81–5.14	<0.001
**Hypertension**	100	103	1.53	1.15–2.03	0.003	1.36	1.01–1.83	0.041
**Functional dependence**	1	22	0.42	0.06–3.05	0.392	0.52	0.07–3.81	0.520

COPD, chronic obstructive pulmonary diseases; NE, no events; RR, relative risk; CI, confidence interval; *Adjusted for all the variables in the table.

**Table 4:** Association between potential predictive factors and death from COVID-19 in the general population cohort.

	Deaths		Sex- and Age-Adjusted Analysis			Fully Adjusted Analysis *		
	*n*	Cases per 100,000	RR	95% CI	*p* Value	RR	95% CI	*p* Value
**Total**	466	72						
**Sex**								
Female	240	74	1			1		
Male	226	71	1.42	1.19–1.71	<0.001	1.61	1.31–1.97	<0.001
**Age, years**								
0–29	0	0	NE			NE		
30-49	2	1	0.06	0.01-0.28	<0.001	0.06	0.01-0.27	<0.001
50-59	16	17	1			1		
60–69	32	45	2.65	1.45–4.83	0.002	2.44	1.33–4.48	0.004
70–79	72	135	8.00	4.65–13.75	<0.001	5.88	3.34–10.34	<0.001
80+	344	920	56.53	34.22–93.37	<0.001	24.43	14.12–42.29	<0.001
**Nursing home resident**	112	2275	5.30	4.25–6.62	<0.001	4.19	3.28–5.36	<0.001
**Healthcare worker**	0	0	NE			NE		
**Place of birth**								
Spain	444	82	1			1		
Europe	1	4	0.25	0.04–1.80	0.169	0.23	0.03–1.67	0.148
Latin America	16	31	2.64	1.59–4.40	<0.001	2.57	1.52–4.36	0.001
North Africa	3	19	1.96	0.63–6.14	0.247	2.03	0.64–6.43	0.230
Sub-Saharan Africa	2	28	3.96	0.97–16.09	0.055	3.41	0.82–14.08	0.090
Other	0	0	NE			NE		
**Place of residence**								
>50,000 inhabitants	147	72	0.87	0.70–1.08	0.203	1.00	0.80–1.25	0.988
5000–50,000 inhabitants	135	61	1.06	0.85–1.32	0.611	1.07	0.86–1.34	0.537
<5000 inhabitants	184	84	1			1		
**Income level**								
Very low	18	64	3.52	2.12–5.86	<0.001	1.95	1.15–3.32	0.013
Low	352	99	1.66	1.31–2.10	<0.001	1.35	1.06–1.72	0.016
Middle	95	37	1			1		
High	1	22	0.65	0.09–4.68	0.671	0.67	0.09–4.79	0.687
**Smoking status**								
Never smoker	29	35	1			1		
Current smoker	200	189	0.77	0.51–1.15	0.202	0.67	0.44–1.01	0.058
Former smoker	47	195	1.08	0.78–1.51	0.643	1.03	0.74–1.44	0.852
Unknown	190	44	0.97	0.79–1.18	0.741	0.80	0.65–0.99	0.039
**Hospitalization in prior year**	88	262	1.72	1.36–2.17	<0.001	1.30	1.02–1.65	0.034
**Immunodeficiency**	8	165	2.74	1.36–5.52	0.005	2.22	1.10–4.48	0.027
**Diabetes**	143	377	1.58	1.29–1.92	<0.001	1.29	1.05–1.58	0.014
**Cardiovascular disease**	172	328	1.52	1.25–1.84	<0.001	1.33	1.09–1.63	0.004
**COPD**	69	249	1.58	1.22–2.05	0.001	1.47	1.12–1.91	0.005
**Asthma**	28	67	1.05	0.72–1.54	0.796	1.03	0.70–1.51	0.886
**Chronic kidney disease**	134	695	1.73	1.41–1.13	<0.001	1.48	1.20–1.83	<0.001
**Cerebrovascular disease**	56	580	1.45	1.09–1.92	0.010	1.04	0.78–1.38	0.803
**Liver cirrhosis**	22	183	1.52	0.99–2.34	0.056	1.37	0.89–2.11	0.156
**Dementia**	83	1669	2.89	2.26–3.69	<0.001	1.56	1.19–2.04	0.002
**Hematological malignancy**	9	333	1.54	0.80–2.98	0.201	1.59	0.82–3.09	0.167
**Non-hematological cancer**	64	165	0.72	0.55–0.93	0.014	0.72	0.55–0.94	0.014
**Severe obesity**	10	119	1.24	0.66–2.33	0.497	0.88	0.47–1.66	0.701
**Hypertension**	314	322	1.36	1.11–1.66	0.003	1.23	1.00–1.51	0.055
**Functional dependence**	95	2073	3.77	2.98–4.76	<0.001	2.24	1.72–2.90	<0.001

COPD, chronic obstructive pulmonary diseases; NE, no events; RR, relative risk; CI, confidence interval; * Adjusted for all the variables in the table.

**Table 5:** Hospitalization, intensive care unit admission and deaths from COVID-19 in hierarchical categories for COVID-19 vaccination priority in the general population cohort (*n* = 643,757). Figures presented are the number, proportion (%) of all events and events per 100,000 inhabitants.

	COVID-19 Hospitalization			Intensive Care Unit Admission by COVID-19			Death from COVID-19		
Categories	*n*	%	Events per 100,000	*n*	%	Events per 100,000	*n*	%	Events per 100,000
**Nursing home resident**	162	7.8	3290	4	1.6	81	112	24.0	2275
**Functional dependent**	86	4.1	2288	1	0.4	27	55	11.8	1463
**≥80 years**									
Chronic conditions	323	15.5	1411	11	4.5	48	171	36.7	747
No chronic conditions	69	3.3	789	3	1.2	34	30	6.4	343
**70–79 years**									
Chronic conditions	232	11.2	741	46	18.7	147	46	9.9	147
No chronic conditions	93	4.5	449	21	8.5	101	11	2.4	53
**60–69 years**									
Chronic conditions	184	8.8	583	39	15.9	123	21	4.5	66
No chronic conditions	152	7.3	391	31	12.6	80	7	1.5	18
**50–59 years**									
Chronic conditions	144	6.9	517	27	11.0	97	7	1.5	25
No chronic conditions	204	9.8	312	31	12.6	47	4	0.9	6
**0–49 years**									
Chronic conditions	106	5.1	162	17	6.9	26	1	0.2	2
No chronic conditions	325	15.6	101	15	6.1	5	1	0.2	0.3
**Total**	2080	100.0	323	246	100.0	38	466	100.0	72

COPD, chronic obstructive pulmonary diseases; NE, no events; RR, relative risk; CI, confidence interval.

## Data Availability

Availability of individual-level data needs authorization of the Department of Health of the Navarra Government.
